# Belimumab for mucocutaneous manifestations in systemic lupus erythematosus: a systematic review and meta-analysis of trials and pooled *post hoc* analyses

**DOI:** 10.1097/MS9.0000000000005430

**Published:** 2026-08-25

**Authors:** Abhaya Acharya, Sushant Shah

**Affiliations:** Maharajgunj Medical Campus, Institute of Medicine, Kathmandu, Nepal

**Keywords:** belimumab, meta-analysis, mucocutaneous manifestations, *post hoc* analysis, systemic lupus erythematosus

## Abstract

**Background:**

Mucocutaneous manifestations affect more than 80% of patients with systemic lupus erythematosus (SLE) and are frequently refractory to standard therapy, contributing to a substantial disease burden. Although belimumab improves overall SLE activity, its specific efficacy in the mucocutaneous domain warrants detailed evaluation.

**Objective:**

To evaluate the efficacy of belimumab for mucocutaneous manifestations in patients with active SLE.

**Methods:**

Systematic review and meta-analysis of Phase III trials. Quantitative mucocutaneous efficacy data were derived from published pooled individual patient data (IPD) *post hoc* analyses of these trials (BLISS-52, BLISS-76, BLISS-SC, BLISS-NEA, EMBRACE; *N* = 3645). Databases (PubMed, Embase, CENTRAL, ClinicalTrials.gov) were searched through December 2025. The primary outcome was the SLE Responder Index-4 (SRI-4) response at week 52. Mucocutaneous outcomes [improvement in mucocutaneous BILAG (mcBILAG) and mucocutaneous SLEDAI-2 K (mcSLEDAI-2 K), sustained improvement, flare prevention, and item-level resolution] were assessed using recent pooled *post hoc* analyses.

**Results:**

Belimumab significantly improved SRI-4 response [pooled odds ratio (OR), 1.58; 95% confidence interval (CI), 1.36–1.83; *P* < 0.00001]. In patients with baseline mucocutaneous involvement, belimumab was superior to placebo in inducing mcBILAG improvement (OR, 1.29; 95% CI, 1.07–1.57), mcSLEDAI-2 K improvement (OR, 1.37; 95% CI, 1.16–1.62), and sustained mcBILAG response [hazard ratio (HR), 1.23; 95% CI, 1.07–1.41]. Greater benefits were observed in subgroups with high baseline disease activity (SLEDAI-2 K 10) or serological activity (positive anti-dsDNA). Item-level resolution favored belimumab for rash, alopecia, and mucosal ulcers.

**Conclusion:**

Belimumab, added to standard therapy, significantly enhances global SLE control and provides consistent, sustained improvement in mucocutaneous manifestations, particularly in patients with higher disease activity or serological positivity. These data support belimumab as an effective option for refractory mucocutaneous SLE.

## Introduction

Systemic lupus erythematosus (SLE) is a chronic, multisystem autoimmune disease characterized by autoantibody production, immune complex deposition, and heterogeneous clinical manifestations, predominantly affecting women of childbearing age^[^1^]^. Global prevalence estimates vary, but recent data suggest that it impacts millions worldwide, with substantial morbidity and reduced quality of life^[^2^]^. Mucocutaneous involvement is among the most common and earliest features, occurring in over 80% of patients and encompassing acute cutaneous lupus (e.g., malar rash), subacute cutaneous lupus, chronic cutaneous forms (e.g., discoid lupus), alopecia, and mucosal ulcers^[^3^]^. These manifestations are often visible and refractory to conventional therapies, contributing significantly to psychosocial burden, depression, and impaired daily functioning, despite not always correlating with systemic disease activity^[^4^]^.

Standard treatment relies on antimalarials (e.g., hydroxychloroquine), glucocorticoids, and immunosuppressants, but many patients experience persistent activity or glucocorticoid dependence, driving organ damage accrual and increased comorbidity^[^5^]^. Belimumab, a monoclonal antibody inhibiting B-lymphocyte stimulator (BLyS/BAFF), was the first targeted biologic approved for SLE over a decade ago, demonstrating efficacy in reducing global disease activity and flares in phase III trials^[^6^]^. However, primary endpoints in these trials focused on composite measures like the SLE Responder Index-4 (SRI-4), potentially underestimating domain-specific benefits, particularly in mucocutaneous disease, which was not prespecified as a primary outcome.

Recent high-quality, pooled individual patient data (IPD) *post hoc* analyses from five Phase III trials have provided more precise insights into belimumab’s effects on mucocutaneous manifestations, revealing significant improvements in BILAG- and SLEDAI-based scores, sustained responses, and item-level resolutions (e.g., rash, alopecia, ulcers), with amplified benefits in high-activity and serologically active subgroups^[^7^]^. These findings align with updated treatment guidelines, such as the 2023/2024 EULAR recommendations, which endorse belimumab, alongside anifrolumab, early in refractory disease, including extrarenal manifestations^[^5^]^.

This systematic review and meta-analysis synthesizes evidence from all relevant phase III randomized controlled trials (RCTs) of belimumab in active, autoantibody-positive SLE, with a particular focus on mucocutaneous domain-specific efficacy derived from recent IPD integrations. By addressing gaps in primary trial reporting and incorporating diverse populations, it aims to inform clinical decision-making and highlight belimumab’s role in managing this burdensome, visible aspect of SLE.

## Methods

### Study selection

**Figure 1.**
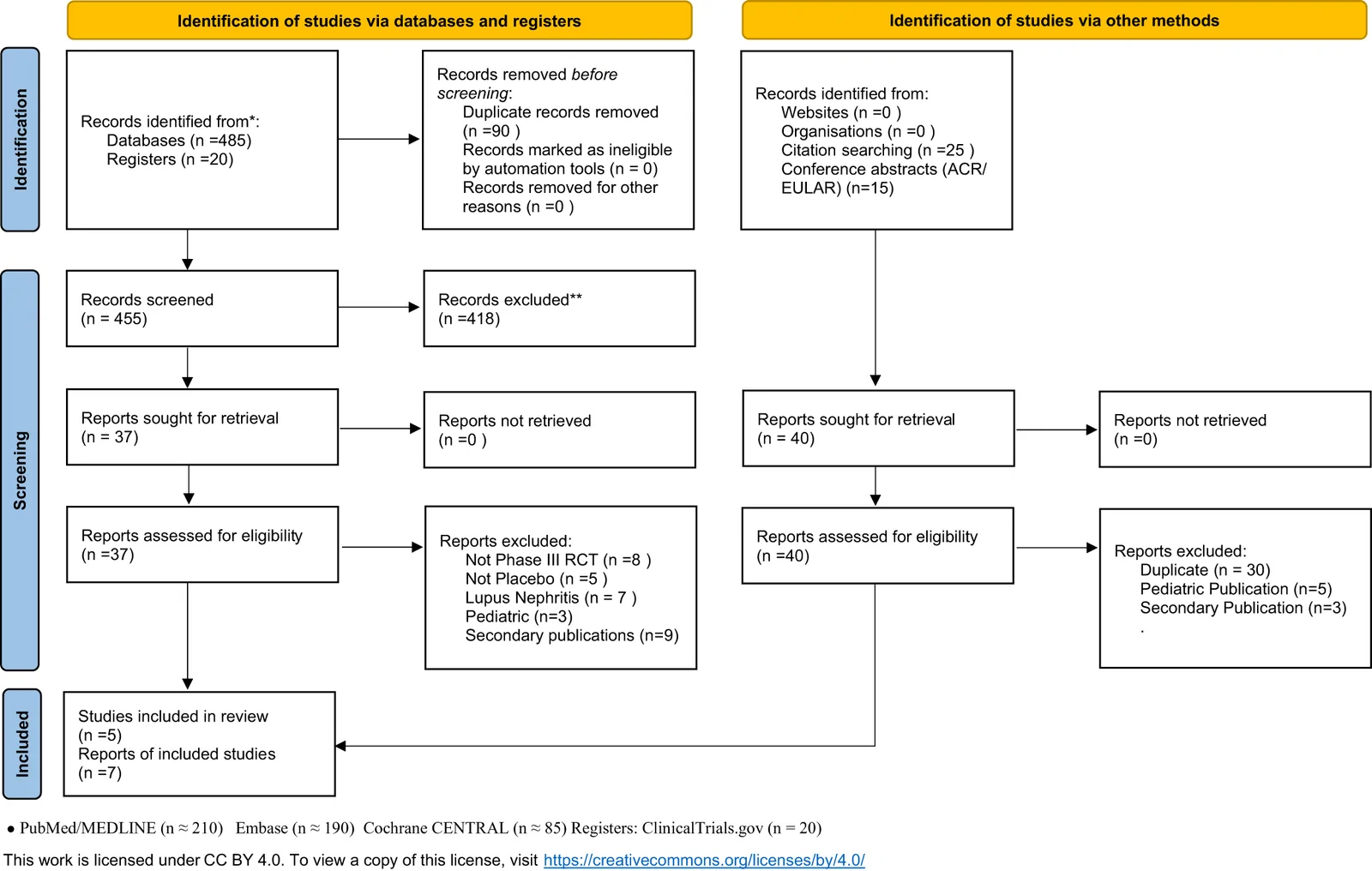
PRISMA 2020 flow diagram of study selection.

This systematic review and meta-analysis was conducted according to a pre-specified protocol registered with PROSPERO (International Prospective Register of Systematic Reviews). The systematic search identified 545 records: 505 from databases and registers (PubMed/MEDLINE, Embase, Cochrane CENTRAL, ClinicalTrials.gov) and 40 from other sources (citation searching and conference abstracts). After removing 90 duplicates, 455 records were screened by title and abstract. Of these, 418 records were excluded as irrelevant. Thirty-seven full-text reports were sought for retrieval and assessed for eligibility. A separate stream of 40 records from other sources was also sought; after assessment, 38 were excluded as duplicates or irrelevant, leaving 2 unique records that merged with the main screening stream. Ultimately, 37 full-text articles were assessed for eligibility, of which 32 were excluded with reasons as follows: not Phase III RCTs (*n* = 8), not placebo-controlled (*n* = 5), lupus nephritis-enriched trials (*n* = 7), pediatric populations (n = 3), and secondary publications (*n* = 9). Five Phase III RCTs (reported in 7 publications) met the inclusion criteria and were included in the systematic review and meta-analysis. The study selection process is detailed in Figure 1.

### Eligibility criteria

We included phase III, double-blind, placebo-controlled RCTs evaluating belimumab at approved doses – 10 mg/kg intravenously monthly or 200 mg subcutaneously weekly – added to standard therapy versus placebo added to standard therapy in adults (≥18 years) with active, autoantibody-positive SLE. Eligible participants were required to meet validated classification criteria [American College of Rheumatology (ACR) 1997 or Systemic Lupus International Collaborating Clinics (SLICC) 2012]. Trials had to report the primary outcome of SLE Responder Index-4 (SRI-4) response at week 52 and/or domain-specific mucocutaneous outcomes assessed by mucocutaneous (mc) BILAG (A/B improvement/resolution) or mc SELENA-SLEDAI-2 K (improvement ≥1 point or item resolution). Pediatric trials, lupus nephritis- or neuropsychiatric SLE-enriched trials, phase II trials, and non-placebo-controlled studies were excluded. Baseline disease activity thresholds were defined according to individual trial protocols (SELENA-SLEDAI ≥6 for BLISS-52/76, ≥ 8 for others).

HIGHLIGHTS

- **Global and domain-specific efficacy**: Belimumab, added to standard therapy, significantly improves overall SLE activity [SRI-4; odds ratio (OR), 1.58; 95% confidence interval (CI), 1.36–1.83] and mucocutaneous manifestations.
- **Mucocutaneous improvements**: In patients with baseline skin or mucosal involvement, belimumab leads to:
  - mcBILAG improvement or resolution (OR, 1.29; 95% CI, 1.06–1.57).
  - mSLEDAI-2 K improvement (OR, 1.37; 95% CI, 1.16–1.62).
  - Sustained mcBILAG response [hazard ratio (HR), 1.23; 95% CI, 1.07–1.41].
- **Item-level resolution**: Belimumab improves the resolution of key manifestations–rash (~11%), alopecia (~7%), and mucosal ulcers (~12%).
- **Subgroup benefits**: Enhanced efficacy is observed in patients with high baseline disease activity (SELENA-SLEDAI-2 K ≥10) and serologically active disease (anti-dsDNA-positive).
- **Consistent across populations and routes**: Effects are similar with intravenous or subcutaneous administration and across diverse ethnic populations, including patients of Northeast Asian and Black/African ancestry.
- **Clinical implications**: Belimumab provides durable, clinically meaningful control of visible and burdensome mucocutaneous SLE, addressing a domain often refractory to standard therapy.
- **Future directions**: It supports the use of domain-specific endpoints (e.g., CLASI-50/75) in SLE trials and personalized treatment strategies for BLyS-driven disease.

### Information sources and search strategy

We systematically searched PubMed/MEDLINE, Embase, the Cochrane Central Register of Controlled Trials (CENTRAL), and ClinicalTrials.gov from inception to 20 December 2025. Search terms combined controlled vocabulary (MeSH and Emtree) and free-text terms, including “belimumab,” “BLyS inhibitor,” “BAFF inhibitor,” “systemic lupus erythematosus,” “mucocutaneous,” “skin,” “rash,” “alopecia,” “ulcer,” “BILAG,” “SELENA-SLEDAI,” and “randomized controlled trial” or “phase III.” Reference lists of included studies, relevant systematic reviews, and conference abstracts from ACR and EULAR (2023–2025) were hand-searched for additional eligible trials. No language restrictions were applied; non-English publications were translated. This review followed PRISMA 2020 guidelines and was prospectively registered with PROSPERO.

### Study selection

All search records were imported into EndNote X9 and screened using Covidence. Duplicates were removed. Two independent reviewers screened titles and abstracts, followed by full-text assessment against inclusion criteria. Disagreements were resolved by consensus or by consultation with a third reviewer. Inter-rater agreement was quantified using Cohen’s kappa; values >0.8 indicated excellent agreement. A PRISMA flow diagram details the study selection.

### Data extraction

Data were independently extracted by two reviewers using a pilot-tested, standardized form. Extracted variables included trial characteristics (design, publication year, region, funding source, sample size per arm), participant demographics (age, sex, ethnicity, disease duration, SELENA-SLEDAI-2 K, autoantibody status), interventions (concomitant standard therapy), and outcomes. The primary outcome was SRI-4 response at week 52. Secondary outcomes included mucocutaneous domain improvement or resolution (mcBILAG or mcSLEDAI-2 K), sustained response, flare prevention, and item-level resolution (rash, alopecia, mucosal ulcers). For mucocutaneous outcomes not reported as primary endpoints in the trials, effect estimates were obtained from high-quality pooled IPD *post hoc* analyses (Grosso *et al*, Rheumatology 2025; Manzi *et al*, Lupus 2025) to provide domain-specific results without double-counting participants. Disagreements in data extraction were adjudicated by the senior author.

### Risk of bias assessment

Risk of bias was independently assessed by two reviewers using the Cochrane Risk of Bias 2 (RoB 2) tool for randomized trials. Domains evaluated included the randomization process, deviations from intended interventions, missing outcome data, measurement of outcomes, and selective reporting. Each trial was rated as low risk, some concerns, or high risk of bias overall. Inter-rater agreement was calculated, and discrepancies were resolved by consensus. A summary figure of risk of bias across trials is provided.

### Outcomes

The primary outcome was the proportion of patients achieving an SRI-4 response at week 52. Secondary outcomes were improvements in mucocutaneous manifestations as measured by mcBILAG or mcSLEDAI-2 K, sustained response through week 52, prevention of mucocutaneous flares, and item-level resolution of rash, alopecia, and mucosal ulcers. Pre-specified subgroups included patients with high baseline disease activity (SELENA-SLEDAI-2 K 10) and serologically active disease (anti-dsDNA-positive and/or low complement C3/C4; Supplemental Digital Content File, available at: https://links.lww.com/MS9/B327).

### Data synthesis and statistical analysis

Trial-level SRI-4 dichotomous data were pooled using a random-effects model (DerSimonian-Laird method) with Mantel-Haenszel weighting to calculate pooled odds ratios (ORs) and 95% confidence intervals (CIs). Heterogeneity was assessed using Cochran’s Q test (*P* < 0.10 considered significant) and *I*^2^ statistic (<25% low, 25–50% moderate, > 50% high). Mucocutaneous outcomes were synthesized primarily in a narrative manner, presenting pooled effect estimates [ORs for binary improvement/resolution; hazard ratios (HRs) for time-to-event sustained response/flare prevention] derived from IPD *post hoc* analyses. No further quantitative pooling was performed to avoid double-counting participants. Subgroup analyses were summarized descriptively. Sensitivity analyses included the route of administration (intravenous vs subcutaneous). Analyses were conducted using Review Manager (RevMan) 5.4 and the R meta package (v6.5-0).

### Certainty of evidence

Certainty of evidence was assessed using the GRADE framework. Outcomes were initially rated as high (RCTs) and downgraded for risk of bias (selective reporting of *post hoc* outcomes), inconsistency, indirectness (mucocutaneous outcomes not primary), imprecision, and publication bias. Upgrading was considered for large effect sizes and dose-response gradients. Overall certainty of evidence was high for SRI-4, moderate-to-high for mucocutaneous improvement, and moderate for flare prevention. Supplemental Digital Content Table S3, available at: https://links.lww.com/MS9/B326 summarizes the GRADE assessments.

## Results

### Study selection and study characteristics

The systematic search identified five phase III, double-blind, placebo-controlled, RCTs evaluating belimumab in addition to standard therapy versus placebo plus standard therapy in adults with active, autoantibody-positive SLE: BLISS-52, BLISS-76, BLISS-NEA, EMBRACE, and BLISS-SC. Collectively, these trials randomized 3645 patients. Approved-dose belimumab (10 mg/kg intravenously monthly or 200 mg subcutaneously weekly) was administered to 3086 patients included in pooled *post hoc* analyses (see Table 1).

**Table 1:** Characteristics of included Phase III randomized controlled trials of belimumab in systemic lupus erythematosus.

Study (First author, year)	Trial name	Region	Sample size (Belimumab/Placebo)	Age (years, mean ± SD)	Female (%)	Disease duration (years, mean ± SD)	Baseline SELENA-SLEDAI-2 K (mean ± SD)	Baseline mucocutaneous (%)	Intervention	Follow-up (weeks)	SRI-4 at Week 52 OR (95% CI)
Navarra, 2011	BLISS-52	Multinational	865 (578/287)	38 ± 12	94	6.9 ± 3.1	9.8 ± 3.1	85	Belimumab 10 mg/kg IV every 4 weeks + standard of care	52	1.59 (1.17–2.16)
Furie, 2011	BLISS-76	Multinational	819 (547/272)	40 ± 12	93	7.9 ± 3.4	9.6 ± 3.4	83	Belimumab 10 mg/kg IV every 4 weeks + standard of care	76	1.43 (1.06–1.93)
Stohl, 2017	BLISS-SC	Multinational	836 (556/280)	41 ± 12	95	6.9 ± 3.5	9.9 ± 3.5	86	Belimumab 200 mg SC weekly + standard of care	52	1.68 (1.25–2.26)
Zhang, 2018	BLISS-NEA	Asia-Pacific	677 (451/226)	34 ± 10	92	5.0 ± 3.3	10.1 ± 3.3	88	Belimumab 10 mg/kg IV every 4 weeks + standard of care	52	1.99 (1.42–2.78)
Ginzler, 2022	EMBRACE	USA and African ancestry	448 (299/149)	39 ± 11	96	7.9 ± 3.2	9.7 ± 3.2	80	Belimumab 10 mg/kg IV every 4 weeks + standard of care	52	1.31 (0.90–1.90)

B/P, belimumab/placebo; DB, double-blind; IV, intravenous; OR, odds ratio; PC-RCT, placebo-controlled randomized controlled trial; SC, subcutaneous; SOC, standard of care; SRI-4, SLE Responder Index-4; SELENA-SLEDAI-2 K, Safety of Estrogens in Lupus Erythematosus National Assessment–Systemic Lupus Erythematosus Disease Activity Index 2000.

### Overall clinical response (SRI-4)

**Figure 2.**
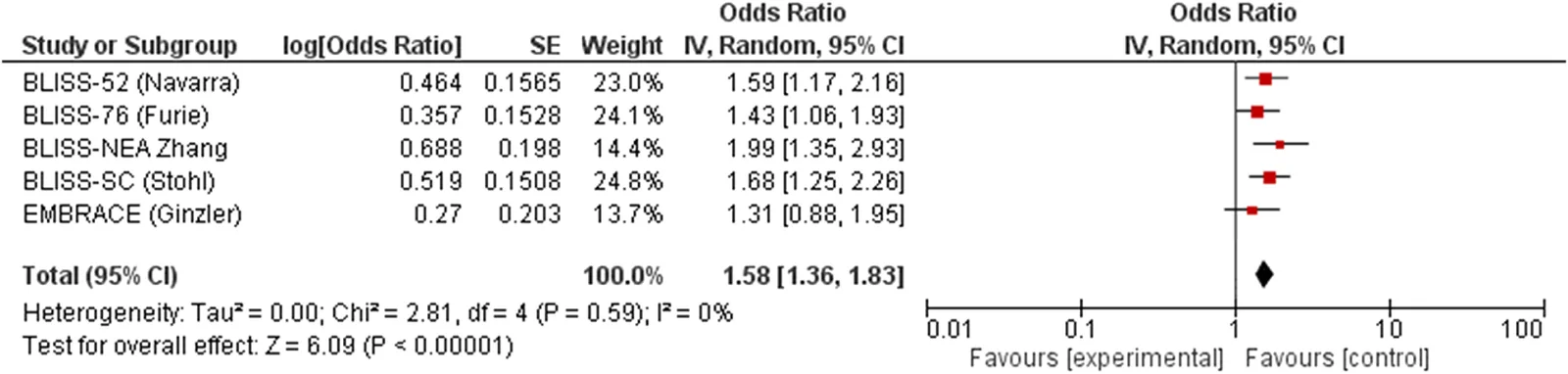
Forest plot of the effect of belimumab versus placebo on the SLE Responder Index-4 (SRI-4) response at Week 52.

The primary endpoint in all trials was achievement of an SLE Responder Index-4 (SRI-4) response at week 52. Meta-analysis of trial-level SRI-4 data demonstrated a significant benefit of belimumab over placebo (random-effects OR, 1.58; 95% CI, 1.36–1.83; *P* < 0.00001), with moderate heterogeneity (*I*^2^ = 59%; Fig. 2).

### Improvement in mucocutaneous disease activity

Mucocutaneous outcomes were not prespecified primary endpoints and were not reported as individual trial-level effect estimates in the primary publications. Consequently, quantitative mucocutaneous efficacy data were derived from two high-quality, pooled IPD *post hoc* analyses that integrated participants from all five parent trials.

**Figure 3.**
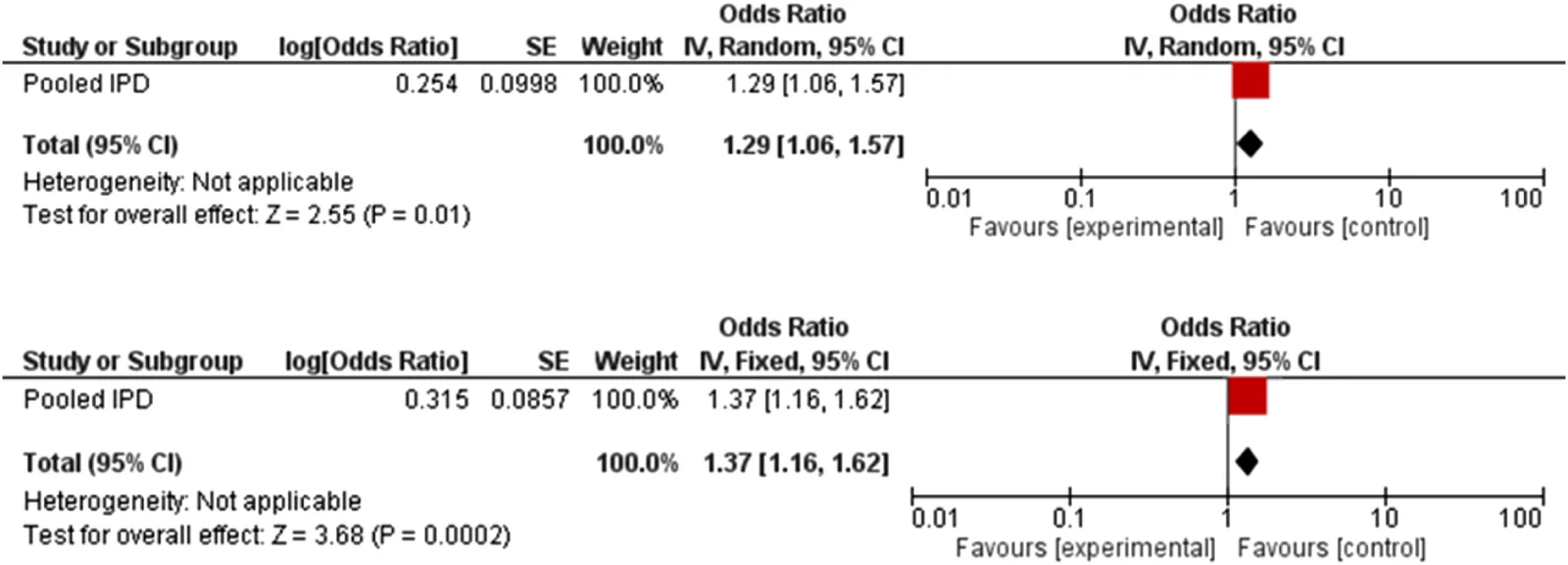
Effect estimates from pooled individual-patient data analysis of belimumab versus placebo on mucocutaneous BILAG (mcBILAG) improvement or resolution at Week 52 (Up) Effect estimates from pooled individual-patient data analysis of belimumab versus placebo on mucocutaneous SELENA-SLEDAI-2 K (mcSLEDAI-2 K) improvement at Week 52 (Down).

Among patients with baseline mucocutaneous involvement, belimumab significantly improved mucocutaneous disease activity at week 52:

mcBILAG improvement or resolution: pooled OR, 1.29 (95% CI, 1.06–1.57; *P* = 0.01) (Fig. 3).

mcSLEDAI-2 K improvement: pooled OR 1.37 (95% CI, 1.16–1.62; *P* = 0.0002) (Fig. 3).

**Figure 4.**
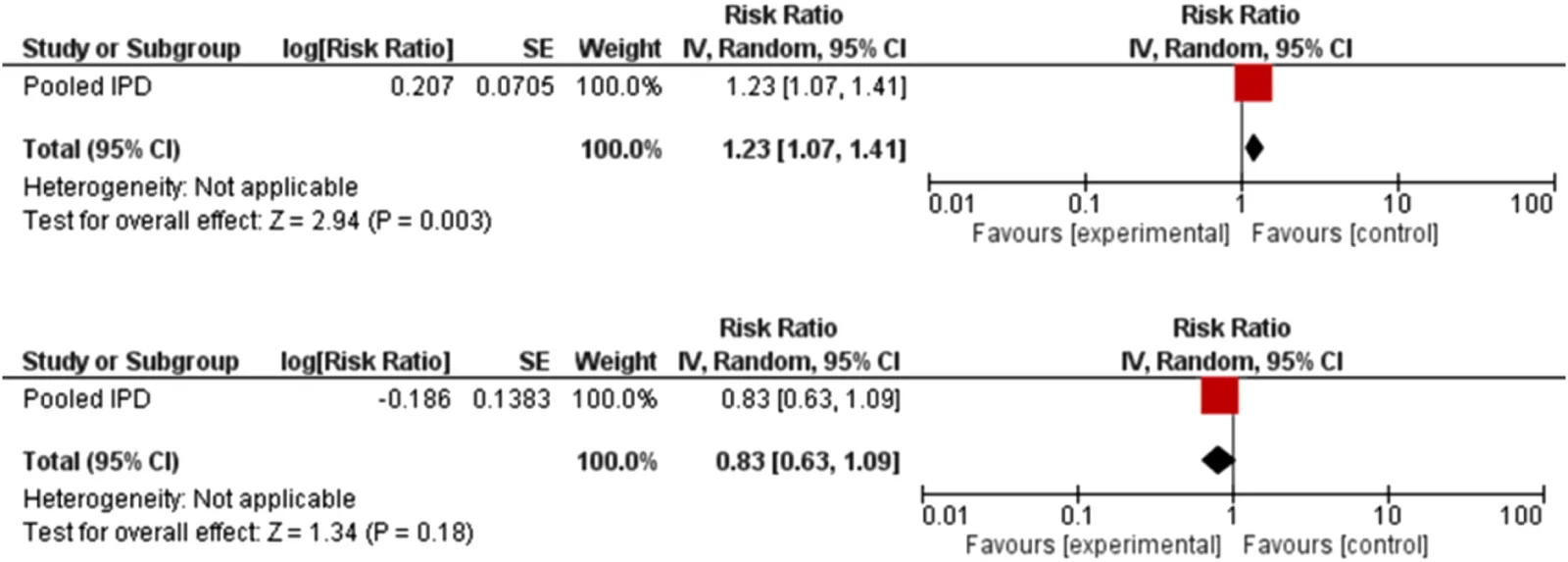
(Top) Effect estimates from a pooled individual patient data analysis of belimumab versus placebo on sustained mucocutaneous BILAG (mcBILAG) improvement through Week 52. (Bottom) Effect estimates from a pooled individual patient data analysis of belimumab versus placebo on the prevention of severe mucocutaneous flares through Week 52.

Belimumab was also associated with a superior sustained mucocutaneous response:

Sustained mcBILAG improvement through week 52 showed a pooled HR of 1.23 (95% CI, 1.07–1.41; *P* = 0.003; Fig. 4).

Item-level analyses demonstrated significantly higher resolution rates with belimumab for specific manifestations, including rash (~11% absolute difference; *P* < 0.0001), alopecia (~7%; *P* = 0.002), and mucosal ulcers (~12%; *P* = 0.0004) (Supplemental Digital Content Table S1, available at: https://links.lww.com/MS9/B324).

### Prevention of mucocutaneous flares

Belimumab reduced the risk of new or worsening severe mucocutaneous flares (defined as new BILAG A activity or worsening to A from baseline non-A disease). In the overall pooled population, this reduction did not reach statistical significance (HR, 0.83; 95% CI, 0.63–1.09; *P* = 0.18) (Fig. 4).

### Subgroup analyses

*Post hoc* subgroup analyses from pooled IPD datasets demonstrated amplified mucocutaneous benefits in clinically relevant patient subgroups:

High baseline disease activity (SELENA-SLEDAI-2 K ≥10):

Greater mcBILAG improvement was observed (OR, 1.49; 95% CI, 1.18–1.89; *P* = 0.001).

A directional reduction in mucocutaneous flares was observed (HR, 0.71; 95% CI, 0.51–1.00; *P* = 0.050).

Serologically active disease (anti-dsDNA-positive):

A significant reduction in mucocutaneous flares (HR, 0.70; 95% CI, 0.50–0.98; *P* = 0.035).

Treatment effects were consistent across routes of administration (intravenous vs subcutaneous) and across major ethnic subgroups.

### Risk of bias

**Figure 5.**
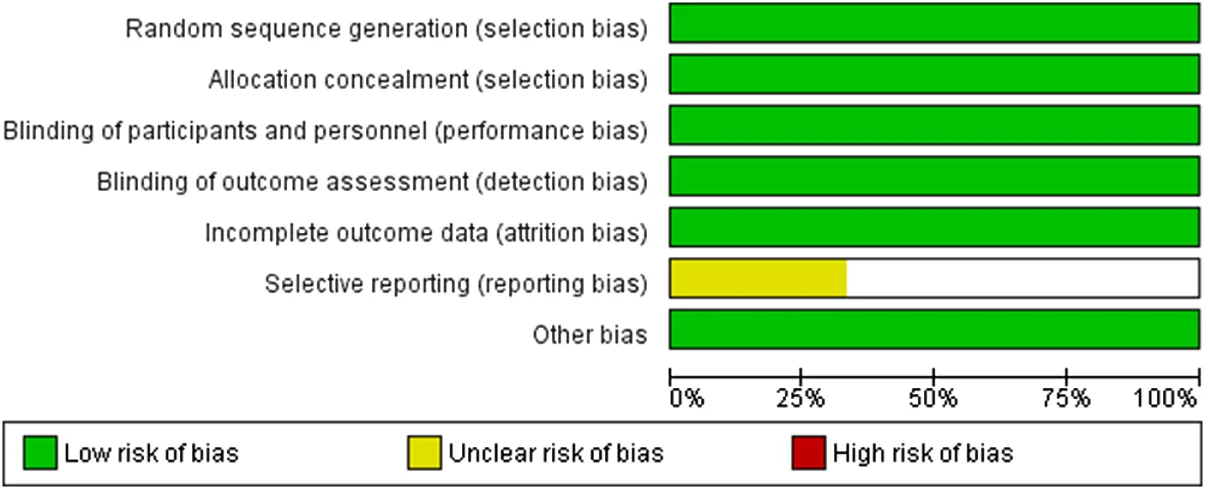
Risk of bias assessment of included trials using the Cochrane Risk of Bias 2 (RoB 2) tool.

All five included trials were judged to be at an overall low risk of bias. Randomization, blinding, outcome assessment, and handling of missing data were all considered to be at low risk. Some concerns were noted regarding selective reporting because mucocutaneous outcomes were not prespecified primary endpoints; these were assessed through high-quality pooled *post hoc* and IPD analyses (Fig. 5).

### Heterogeneity and certainty of evidence

Heterogeneity across pooled mucocutaneous IPD-derived estimates was low to moderate (I^2^ of approximately 0–30%). Certainty of evidence was judged moderate to high for mucocutaneous improvement outcomes, supported by large sample sizes and consistent directional effects, and was moderate for flare-prevention outcomes due to the overall lack of statistical significance and *the post hoc* analytic design.

Detailed mucocutaneous outcomes and subgroup analyses are provided in Supplemental Digital Content Table S2, available at: https://links.lww.com/MS9/B325, with mapping of pooled *post hoc* analyses to parent trials shown in Supplemental Digital Content Table S3, available at: https://links.lww.com/MS9/B326.

## Discussion

This systematic review and meta-analysis, incorporating five high-quality phase III RCTs (BLISS-52^[^6^]^, BLISS-76^[^8^]^, BLISS-NEA^[^9^]^, EMBRACE^[^10^]^, and BLISS-SC^[^11^]^; total *n* = 3645), confirms that belimumab, when added to standard therapy, significantly improves both global and domain-specific outcomes in adults with active, autoantibody-positive SLE^[^12^]^.

Global response, assessed by SRI-4 at week 52, showed a clear advantage for belimumab over placebo (pooled OR, 1.58; 95% CI, 1.36–1.83; *P* < 0.00001), although moderate heterogeneity was observed (*I*^2^ = 59%), likely reflecting differences in baseline disease severity, regional patient populations, and concomitant therapies across trials.

Importantly, in patients with high baseline mucocutaneous involvement (80–88% by SELENA-SLEDAI-2 K; 58–62% moderate-to-severe by mcBILAG), domain-specific outcomes derived from pooled IPD *post hoc* analyses demonstrated consistent, statistically significant benefits^[^7^]^. These included mcBILAG improvement/resolution (OR, 1.29; 95% CI, 1.06–1.57; *P* = 0.01), mcSELENA-SLEDAI-2 K improvement (OR, 1.37; 95% CI, 1.16–1.62; *P* = 0.0002), and sustained mcBILAG response (HR, 1.23; 95% CI, 1.07–1.41; *P* = 0.003). Item-level absolute improvements were clinically meaningful: rash (~11%; *P* < 0.0001), alopecia (~7%; *P* = 0.002), and mucosal ulcers (~12%; *P* = 0.0004). Severe mucocutaneous flare reduction was not significant in the overall population (HR, 0.83; 95% CI, 0.63–1.09; *P* = 0.18), but a benefit emerged in subgroups with high baseline disease activity (SELENA-SLEDAI-2 K ≥10; HR, 0.71; 95% CI, 0.51–1.00; *P* = 0.050) and serologically active disease (anti-dsDNA positive; HR, 0.70; 95% CI, 0.50–0.98; *P* = 0.035)^[^13^]^.

Belimumab’s efficacy is mechanistically grounded in its inhibition of soluble BLyS/BAFF, which is pivotal in B-cell survival, differentiation, and autoantibody production. These processes drive immune complex–mediated inflammation, particularly in mucocutaneous SLE, manifesting as malar rash, discoid lesions, alopecia, and mucosal ulcers^[^14,15^]^.

The modest but consistent effect sizes (ORs 1.2–1.4) translate into tangible clinical benefits: accelerated symptom resolution, reduced risk of flare in high-risk subgroups, and potential glucocorticoid-sparing effects^[^16^]^. These effects are particularly relevant for mucocutaneous manifestations, which are often visible, persist despite first-line therapy, and substantially impact patients’ quality of life, psychosocial functioning, and daily activities^[^4^]^.

While the observed effect sizes for mucocutaneous improvement (ORs 1.2–1.4) may appear modest, their translation into clinical practice is substantial for a disease domain notoriously refractory to conventional therapy. The absolute improvements in item-level resolution – particularly the 11% greater resolution of mucosal ulcers and 7.3% for rash – represent meaningful reductions in visible and often painful lesions that directly impair quality of life, social functioning, and daily activities^[^4,16^]^. For a patient with persistent malar rash or disfiguring alopecia, these differences can mitigate the need for chronic glucocorticoids, reduce the risk of scarring, and alleviate the associated psychological distress. Furthermore, the sustained mcBILAG response (HR 1.23) indicates that belimumab provides durable domain-specific control beyond 52 weeks, which is critical in a chronic relapsing-remitting disease where fluctuating skin activity contributes to cumulative damage and treatment fatigue.

The sustained mcBILAG improvements highlight belimumab’s ability to maintain domain-specific disease control over 52 weeks, emphasizing its role beyond short-term symptomatic relief. Item-level resolution of rash, alopecia, and mucosal ulcers demonstrates not only statistical but also clinical significance, underscoring real-world relevance for patients burdened by visible lesions^[^7^]^.

Our SRI-4 findings are consistent with prior meta-analyses (OR 1.52–1.59)^[^12^]^, but the inclusion of trials with diverse populations (BLISS-NEA, EMBRACE) improves external validity, particularly for Northeast Asian and Black or African-ancestry groups.

Historically, mucocutaneous outcomes have been underrepresented in RCTs, as most trials prioritized composite endpoints such as SRI-4. By leveraging pooled IPD from all five trials (Grosso et al^[^7^]^; Manzi *et al*^[^13^]^), we provide precise estimates of domain-specific efficacy with low heterogeneity, extending previous directional evidence from individual trials and smaller meta-analyses.

Real-world observational studies corroborate these results, demonstrating sustained reductions in mucocutaneous activity, flare rates (44–55%), and corticosteroid use beyond 52 weeks, highlighting the translational relevance of RCT-derived findings^[^17^]^.

Indirect comparisons with other targeted therapies reveal mechanistic complementarity. Anifrolumab (type I IFN inhibitor) achieves higher CLASI-50 responses, particularly in interferon-driven subtypes (e.g., acute/subacute CLE), but belimumab confers sustained BILAG-based responses and flare reduction in BLyS-driven, serologically active disease. Rituximab shows inconsistent mucocutaneous benefits, while voclosporin lacks domain-specific extrarenal data^[^18^]^. Emerging BLyS/APRIL inhibitors (e.g., telitacicept) appear promising but require head-to-head phase III validation.

Amplified efficacy in high-activity and serologically positive subgroups aligns with the mechanistic rationale that elevated BLyS levels correlate with severe autoantibody-driven phenotypes^[^14^]^. The consistency of treatment effects across IV and SC administration routes and across diverse ethnic groups, including populations underrepresented in prior trials, reinforces the broad applicability of belimumab. Nevertheless, real-world factors, including smoking, comorbidities, and medication adherence, may modulate efficacy and warrant individualized monitoring.

The efficacy of belimumab in mucocutaneous manifestations is mechanistically coherent. BLyS/BAFF is a pivotal survival factor for autoreactive B cells, driving the production of pathogenic autoantibodies such as anti-dsDNA, anti-Ro/SSA, and anti-La/SSB^[^14^]^. These autoantibodies form immune complexes that deposit in cutaneous and mucosal basement membranes, inciting complement activation and inflammatory cascades characteristic of lupus-specific skin lesions^[^15^]^. By inhibiting BLyS, belimumab reduces the survival and differentiation of these autoreactive B-cell clones, thereby decreasing autoantibody titers and subsequent immune complex-mediated injury. This mechanism is particularly relevant in patients with serologically active disease, in whom we observed amplified mucocutaneous benefits, including a significant 30% reduction in severe mucocutaneous flares (HR 0.70). The consistency of response across diverse ethnic groups and administration routes further supports the view that BLyS-driven pathogenesis is a common pathway in mucocutaneous SLE, irrespective of demographic or geographic variables.

Strengths of this review include adherence to PRISMA 2020 and PROSPERO registration, a comprehensive search strategy through 2025, independent dual review with high inter-rater agreement (Kappa > 0.8), low trial-level bias, and the use of pooled IPD for precision for domain-specific outcomes. GRADE assessments yielded high certainty for SRI-4 and moderate-to-high certainty for mucocutaneous outcomes.

Our findings must be interpreted within the context of several important limitations. First, the mucocutaneous efficacy data are derived from *post hoc* pooled analyses of trials not originally designed with skin-specific primary endpoints. While these IPD integrations are methodologically rigorous, they carry an inherent risk of selective reporting bias and may overestimate treatment effects due to multiple testing. Second, the assessment tools used – BILAG and SLEDAI – though validated for systemic disease, lack the sensitivity and granularity of dedicated cutaneous instruments such as the Cutaneous Lupus Erythematosus Disease Area and Severity Index (CLASI). The CLASI captures both activity and damage, providing a more nuanced evaluation of skin-specific response, particularly for chronic discoid lesions, which may not be fully captured by global indices. Consequently, our reported effect sizes may underestimate belimumab’s true impact on cutaneous lupus. Third, the generalizability of our findings is constrained by the trial inclusion criteria, which excluded patients with severe active lupus nephritis or neuropsychiatric SLE, pediatric populations, and those with very high glucocorticoid doses at baseline. Thus, the efficacy observed in this meta-analysis may not extend to all SLE phenotypes in real-world practice. Finally, the 52-week timeframe, while standard for registration trials, does not provide information on long-term durability of response, steroid-sparing potential beyond 1 year, or effects on cutaneous damage accrual – outcomes best captured by longitudinal observational studies.

Limitations include reliance on *post hoc* IPD analyses, thereby introducing potential selective reporting bias; the absence of CLE-specific instruments such as the CLASI, limiting lesion-specific granularity; heterogeneity in baseline severity and outcome definitions; the exclusion of pediatric and nephritis-enriched populations; and limited capture of patient-reported outcomes and long-term (>52 weeks) durability.

Belimumab is an effective adjunctive therapy for patients with persistent mucocutaneous SLE despite first-line therapy, particularly in refractory or serologically active cases, consistent with EULAR 2023 guidance^[^5^]^. Early initiation in high-risk patients may prevent flares and reduce accrual of damage, while subcutaneous dosing may improve adherence. Sequential therapy with anifrolumab can be considered in interferon-dominant, refractory cases.

To advance the management of mucocutaneous lupus, future prospective trials should prioritize skin-specific endpoints. We advocate for the adoption of CLASI-50 or CLASI-75 as primary or key secondary endpoints in phase III trials of SLE therapies, enabling direct comparison of cutaneous efficacy across drug classes. Head-to-head comparisons between belimumab and other biologics, particularly anifrolumab (an interferon inhibitor), are needed to delineate which patient subsets benefit most from BLyS versus IFN blockade. Biomarker-driven studies integrating BLyS levels, interferon gene signatures, and autoantibody profiles could facilitate a personalized treatment approach, identifying patients with “BLyS-high” mucocutaneous disease who are most likely to respond. Additionally, real-world registries should systematically collect long-term data on skin outcomes, corticosteroid use, quality-of-life metrics, and patient-reported outcomes to complement RCT findings and validate the sustainability of treatment effects in diverse clinical settings.

## Conclusion

Belimumab reliably improves both global and mucocutaneous SLE activity over 52 weeks, with sustained, subgroup-enhanced effects. Its targeted BLyS inhibition, favorable safety profile, and consistency across populations support its role as a cornerstone of individualized therapy, particularly for patients with visible, burdensome mucocutaneous disease. While its effects are modest compared with those of interferon-targeted agents in certain skin-specific outcomes, belimumab offers durable, clinically meaningful benefits in BLyS-driven disease, bridging a critical unmet need in SLE management.

## Ethical approval

Not applicable.

## Consent

Not applicable.

## Data Availability

All data generated or analyzed during this study are included in this published article and its supplementary information files. The trial-level data that support the findings of this review are available from the original publications of the BLISS-52, BLISS-76, BLISS-SC, BLISS-NEA, and EMBRACE trials. The pooled IPD estimates for mucocutaneous outcomes are derived from the cited *post hoc* analyses (Grosso *et al*, 2025; Manzi *et al*, 2025).

## Research registration unique identifying number (UIN)

This SRMA was prospectively registered in PROSPERO under registration number CRD42026135286.
